# Health financing challenges in Southeast Asian countries for universal health coverage: a systematic review

**DOI:** 10.1186/s13690-023-01159-3

**Published:** 2023-08-17

**Authors:** Ming Yao Lim, Hanin Farhana Kamaruzaman, Olivia Wu, Claudia Geue

**Affiliations:** Health Economics and Health Technology Assessment, School of Health and Wellbeing, University of Glasgow, Scotland, UK

**Keywords:** Health Financing, Health Economics, Universal Health Care, ASEAN

## Abstract

**Background:**

Universal Health Coverage (UHC) has received much attention and many countries are striving to achieve it. The Southeast Asian region, in particular, comprises many developing countries with limited resources, exacerbating challenges around attaining UHC. This paper aims to specifically explore the health financing challenges these countries face in achieving UHC via a systematic review approach and formulate recommendations that will be useful for policymakers.

**Methods:**

The systematic review followed the guidelines as recommended by PRISMA. The narrative synthesis approach was used for data synthesis, followed by identifying common themes.

**Results:**

The initial search returned 160 articles, and 32 articles were included after the screening process. The identified challenges in health financing towards achieving UHC in the Southeast Asian region are categorised into six main themes, namely (1) Unsustainability of revenue-raising methods, (2) Fragmented health insurance schemes, (3) Incongruity between insurance benefits and people’s needs, (4) Political and legislative indifference, (5) Intractable and rapidly rising healthcare cost, (6) Morally reprehensible behaviours.

**Conclusions:**

The challenges identified are diverse and therefore require a multifaceted approach. Regional collaborative efforts between countries will play an essential role in the progress towards UHC and in narrowing the inequity gap. At the national level, individual countries must work towards sustainable health financing strategies by leveraging innovative digital technologies and constantly adapting to dynamic health trends.

**Registration:**

This study is registered with PROSPERO, under registration number CRD42022336624.

**Supplementary Information:**

The online version contains supplementary material available at 10.1186/s13690-023-01159-3.

**Table:** 

**Text box 1.** Contributions to the literature
• A multitude of political, economic, moral, legal, epidemiological and bureaucratic factors is hindering the progress towards UHC within the ASEAN region. These issues are complex and often intertwined; therefore, they should be addressed by harmonising governmental efforts and national policies.
• There is untapped potential in exploring innovative financing schemes to narrow health and wealth inequality gaps. Investing in digital health technologies and developing research capacity for strategies to tackle ageing populations and transiting demographics should be considered. A list of potential recommendations for policymakers is provided in this paper.

## Background

Universal Health Coverage (UHC) was first conceptualised by the World Health Organisation (WHO) during the fifty-eighth assembly [1]. It is defined as “*a system in which everyone in a society can get the health-care services they need without incurring financial hardship*.” [2] UHC has three important goals: providing financial protection from the costs of using health services, ensuring equity in the use of health services and maintaining the quality of health services [3]. While achieving these three goals is challenging, UHC is the cornerstone to achieving better health outcomes [4]. Health financing is necessary to source funding for health services as well as determining the optimal structure for the allocation of these resources [5]. It is important to recognise that there is no “gold standard” when it comes to health financing for UHC [1] because every country has its own set of unique political, cultural, social and economic circumstances.

The health-finance-related challenges relating to UHC are diverse. During the 2014 OECD meeting, the importance of sustainable healthcare financing was acknowledged. The sustainability of government revenue-based health financing is susceptible to economic impact and magnified by globalisation which widens the economic inequalities between countries [6]. In international efforts to narrow this gap, a global equalisation scheme proposed by Sustainable Development Solutions Network (SDSN) suggests high-income countries contribute 0.1% of their Gross Domestic Product (GDP) to international health assistance [7]. Ooms et al. [8] questioned if such a global effort would be a practical solution toward achieving a minimum domestic public health financing. The SDSN proposal is financially demanding for developing countries, requiring government health expenditure to increase from 1.5 to 3.25% of the GDP. While this might be possible for developed countries, it would be an arduous journey for low-income countries, highlighting the differential consequences of the underlying income inequity between developing and developed nations.

The Association of Southeast Asian Nations, also known as ASEAN, was established in 1967 by Indonesia, Malaysia, the Philippines, Singapore and Thailand. Subsequently, Brunei, Cambodia, Laos, Myanmar and Vietnam joined the association, forming the ten ASEAN member states today. Since the ASEAN region is diverse in social, cultural, economic and political aspects, it also features a wide variety of healthcare systems [9–12] and health financing approaches [11].

The health status of the Southeast Asia region has a tremendous influence on global health. ASEAN countries like Indonesia and Thailand have made good progress towards achieving UHC [10], and other nations can emulate their success. This region has a good representation of countries from various economies, including high-, lower-middle and upper-middle-income economies [13]. Our focus on health financing, which is strongly connected to a nation’s economic performance, coupled with the heterogeneity in ASEAN countries regarding the economy, grants a broader perspective.

There is only one published systematic review that studies health financing issues within the ASEAN region [14]. It discusses the impact of healthcare financing mechanisms on the UHC goals but does not touch on the challenges involved. In a narrative article published in 2011 [9], the existing healthcare financing systems in ASEAN countries were elaborated on briefly alongside their recent development. A slightly more technical paper written from the perspective of financing explored the effect of fiscal capacity on the healthcare financing policy in the Southeast Asian region [10]. However, none of the studies above specifically addressed the challenges faced in health financing issues en route to achieving UHC.

Lagarde and Palmer [15] published a protocol in the Cochrane Review to assess the effectiveness of healthcare financing in low- and middle-income countries. Stringent details following the Cochrane review process were specified, including the bibliographic databases, search terms, data collection, data analysis and quality assessment. Subsequently, three papers were published [16–18] following the application of the explicitly stated methods in the protocol. An overview of systematic reviews on financial arrangements for health systems in low-income countries was published in 2017 [19]. However, these articles generally have a broader scope of low- and middle-income countries and do not focus on the ASEAN region.

This systematic review aimed at addressing the gaps in evidence in current literature by (i) identifying the challenges of healthcare financing systems of ASEAN countries to achieving UHC and (ii) developing a set of recommendations based on the challenges identified.

## Methods

The systematic review was conducted in June 2022 according to the criteria in the Preferred Reporting Items for Systematic Review and Meta-Analyses (PRISMA) guidelines. It was registered with PROSPERO under registration number CRD42022336624.

### Search Strategy

Five databases were searched: CINAHL, Medline, EMBASE, PubMed and EconLit. The search was divided into several domains, and the corresponding keywords were used to capture the concept reflected in each domain (Table 1). The detailed search strategy is available in Appendix 1.

**Table 1:** List of domains and keywords for the search

No	Domain	Keywords
**1**	Barriers	“barriers” OR “issues” OR “obstacles” OR “difficulties” OR “problems” OR “challenges” OR “lessons” OR “experiences”
**2**	Healthcare finance	“health finance” OR “healthcare finance” OR “health expenditure” OR “healthcare expenditure” OR “health insurance system”
**3**	Universal Health Coverage	“universal health coverage” OR “universal health care” OR “UHC” OR “universal coverage” OR “utility” OR “equity”
**4**	ASEAN countries	“ASEAN” OR “Southeast Asia” OR “Brunei” OR “Brunei Darussalam” OR “Cambodia” OR “Indonesia” OR “Lao PDR” OR “Lao” OR “Malaysia” OR “Myanmar” OR “Philippines” OR “Singapore” OR “Thailand” OR “Vietnam”

### Inclusion and exclusion criteria

Only articles that covered all four domains, as outlined in Table 1, were included. In addition, several restriction criteria were applied: (1) Peer-reviewed publications, (2) Papers published in the English language, and (3) Publications dating from the year 2010 onwards. Implementation of healthcare policies takes a considerable amount of time, and a further period is required to appreciate the effects of these policies. In order to appraise the most recent and relevant evidence, the year 2010 was taken as the starting point. The search was not limited to any particular type of study design. Papers were excluded from the review if the full article was not retrievable despite the authors’ best efforts.

### Study selection, data extraction and synthesis

Screening for full eligibility was done using a criteria checklist corresponding to the inclusion and exclusion criteria as stated above. Records identified from all five databases were checked for duplication using the automatic tool embedded in Endnote and manually by LMY. Subsequently, all records were screened in two stages, by title and by abstract content. The remaining articles were then retrieved in full and assessed for eligibility. A data extraction form was created according to the recommendations outlined by Buchter et al. [20]. To optimise data extraction, piloting the data extraction form was carried out, followed by some modifications. The final version consisted of details including the first author, journal, year, objective(s), methods, country(s), data source, main results, health-financing-related challenges, source of funding and conflict of interest.

**Fig. 1:**
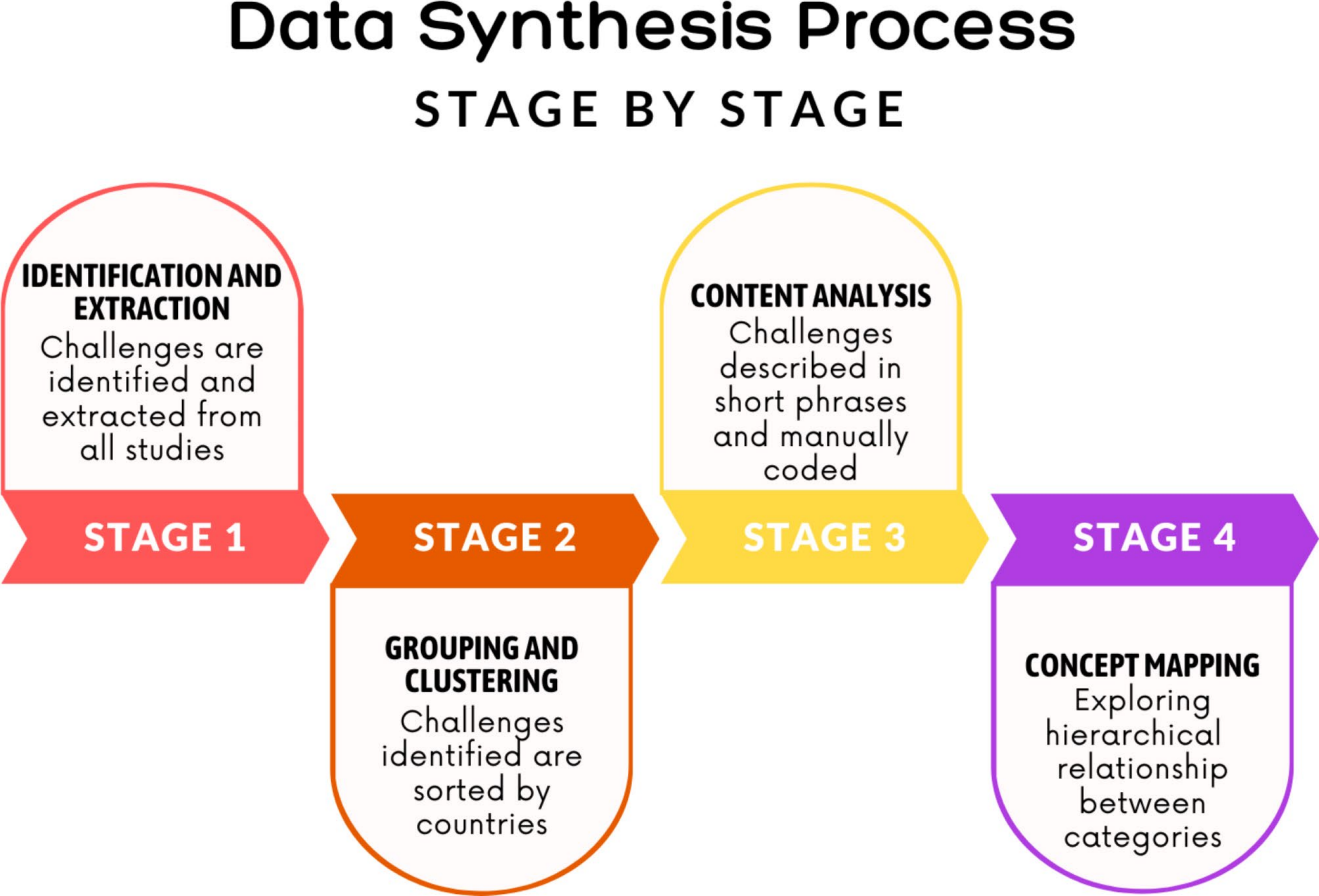
Summary of the Data Synthesis Process

The data synthesis process was adapted from the narrative synthesis guidelines for systematic reviews [21]*(*Fig. 1*).* In the first stage, all health finance challenges related to achieving UHC were identified and extracted from all studies. The second stage involved grouping and clustering the challenges. Similar challenges representing the same idea are identified and summarised within the same group. Subsequently, groups are organised into clusters based on their country. The third stage was content analysis [22], where the challenges were described in short phrases succinctly and collapsed into fewer categories where possible. The last stage was concept mapping, where relationships between categories were explored. Concept mapping was done by visualising the categories with a flowchart and examining any potential relationship between them.

### Quality assessment of included studies

The quality assessment checklist from the Critical Appraisal Skills Programme (CASP) website was used to appraise systematic reviews and qualitative studies [23]. However, the appraisal checklist [24] from Joanna Briggs Institute (JBI) was used for cross-sectional analytical studies. LMY reviewed all articles, and a randomly selected 40% of the articles were reviewed by CG and HFK. The review processes were carried out independently.

## Results

**Fig. 2:**
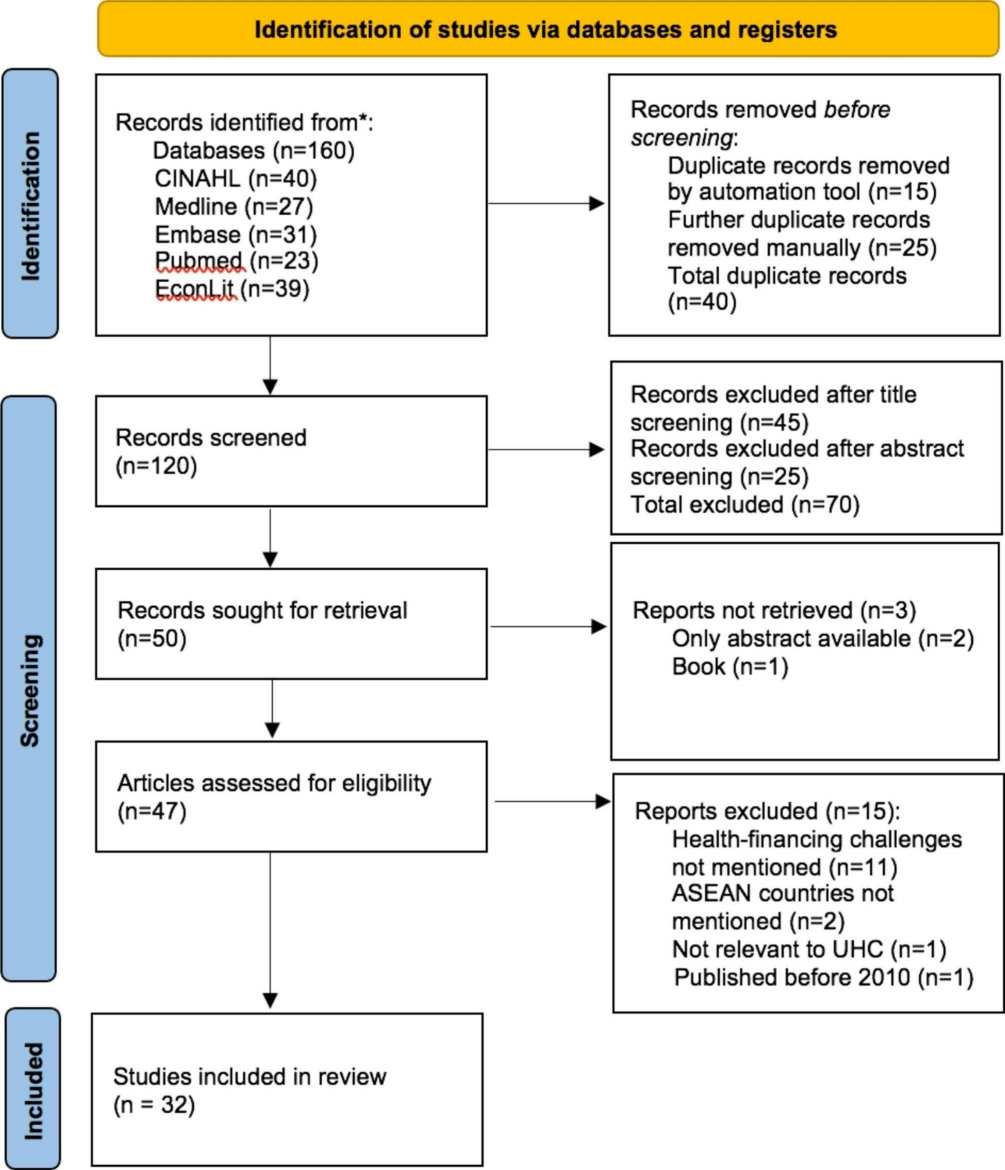
PRISMA Flow Diagram

**Fig. 3:**
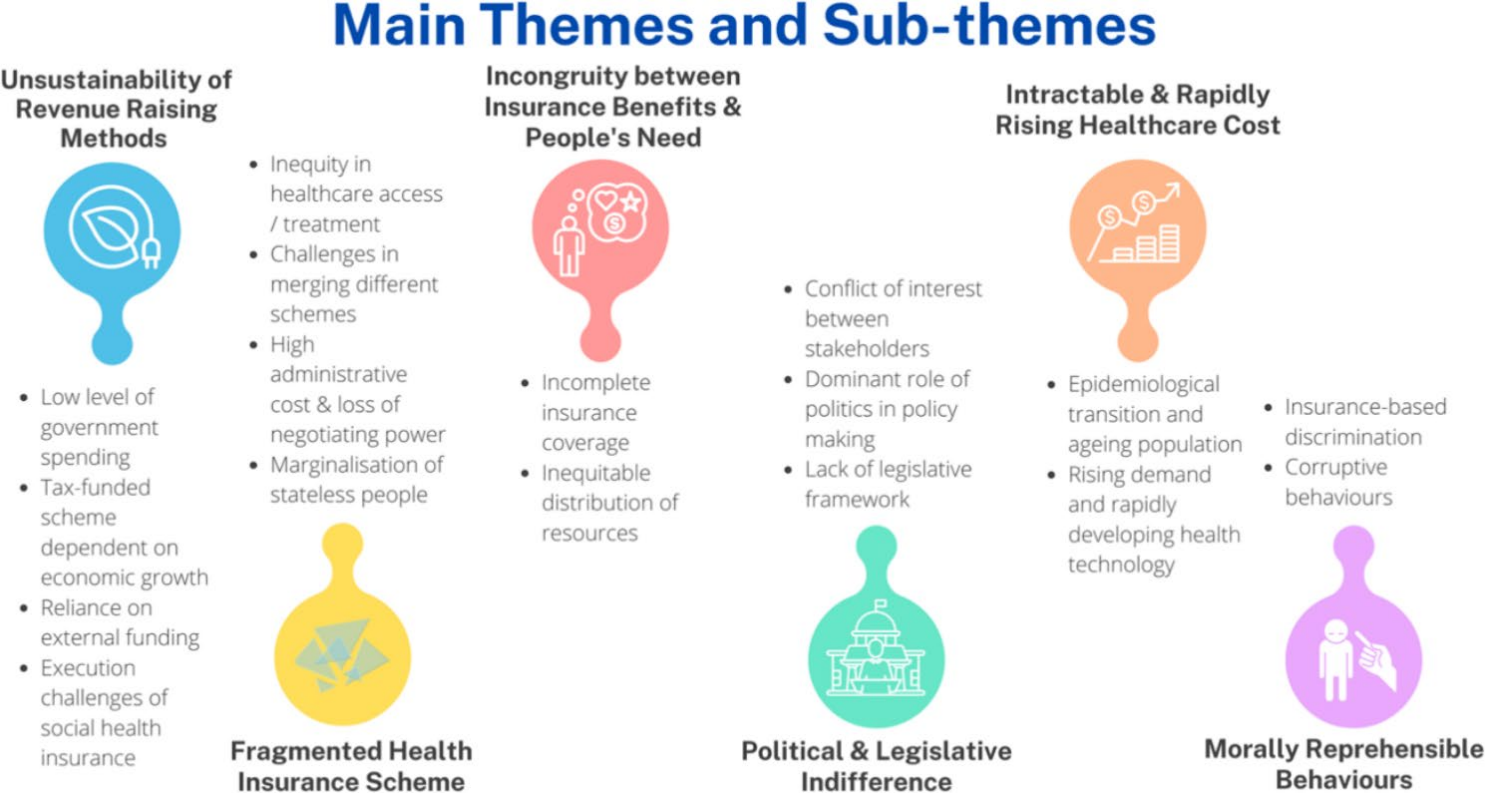
Summary of Main Themes and Sub-themes

A total of 160 records were identified from the search, and 32 articles were included after title and abstract screening *(*Fig. 2*).* There were two systematic reviews [14, 25], 11 scoping review articles [26–36], seven qualitative articles [37–43], and six cross-sectional analytical articles [44–49]. The remaining six articles did not conform to any of those categories and involved a variety of techniques [50–55], including mixed methods. No articles focused specifically on Brunei, Laos, Singapore or Myanmar. A summary of evidence extracted from the articles reviewed is available as a supplementary file in Appendix 2. Following data synthesis, six main overarching themes were identified *(*Fig. 3*).* Most of these themes are common to lower-middle-income and upper-middle-income countries (LMIC and UMIC). Each main theme consists of related sub-themes, which are further explored.

### I) Unsustainability of revenue-raising methods

The elements that make up the unsustainability of revenue-raising methods according to our study are as follows:

#### • Low level of government spending

According to the statistics provided by WHO in 2010, in Malaysia, the Philippines, Indonesia, Vietnam and Laos, the government budget allocated to health is less than 9% of the total budget [32]. Low government healthcare expenditure was found to be a barrier to achieving UHC as vulnerable populations are heavily dependent on public financing [14]. Inadequate government spending in Indonesia is reported in several articles [33, 35, 36]. A similar situation is also present in Thailand, where the hospital operating cost from the government budget has been decreasing steadily [46, 52]. This chronic underfunding leads to a rising shift of costs to providers and patients, contributing to medical impoverishment [29, 52] and retards progress towards UHC.

#### • Tax-funded scheme dependent on economic growth

A predominantly tax-funded scheme is mainly dependent on economic performance [55]. In Thailand, poor economic performance might diminish the government tax revenue and questions regarding the long-term sustainability of the health finance system arise [25, 27, 48, 51]. Given the cyclical nature of the economy, the inconsistent government tax revenue may not match the growing healthcare cost and hence jeopardise the sustainability of the health finance system.

#### • Reliance on external funding

In Laos and Cambodia, large sums of funding originate from external donors (14.5% and 16.4% of the total health expenditure, respectively) [32]. The long-term sustainability is questionable since the autonomy of the external funding does not lie with the government itself. Furthermore, the national health needs of the country might not align with the donor-funded programmes. A similar pattern is found in the Philippines, where the government subsidies for health care did not come from the regular budget but from third parties [41]. Lack of control of funding sources is a significant obstacle to health finance planning.

#### • Execution challenges of social health insurance

There are numerous challenges concerning implementing social health insurance (SHI) schemes. A sizeable informal worker sector hinders Thailand’s premium collection process as it is technically unfeasible [31, 32, 48, 52, 56]. In addition to high administrative costs in Vietnam, it is also difficult to reach rural areas where approximately 60% of the informal sector workers reside [32, 37, 42]. The same issue is observed in both Cambodia and the Philippines [32, 50]. Even for those who managed to enrol, the long waiting period between payment and receiving the health insurance card might undermine the effectiveness of SHI [38].

Indonesia faces a similar predicament as the local authority struggles to seek a cost-effective way to collect premiums [49]. Income instability, inflexibility in the payment system, unaffordable premium rates, and prioritising other essential expenditures over health insurance are causes for deferring premium payments [36, 49]. In addition to jeopardising the long-term financial viability of the SHI schemes, a low enrolment rate ensues [35, 36]. There are also gaps between SHI enrolment and actual utilisation of health facilities [49]. The development of SHI tailored to the needs of the local community can be time-consuming [38].

The lack of health insurance literacy in Cambodia and Indonesia has contributed to the unwillingness to pay for SHI premiums [49, 50]. In Vietnam, many informal sector workers perceive themselves as having low health risks and therefore deem SHI unnecessary [42].

### ii) Fragmented health insurance schemes

The components identified in our research that contribute to fragmented health insurance schemes are listed below:

#### • Inequity in healthcare access/treatment

Fragmented health insurance schemes limit the potential degree of risk distribution due to inequitable healthcare access or treatment [35]. In Vietnam, the insurance coverage at a community health centre is lower than at district-level hospitals [14]. Due to differences in health insurance schemes and capitation payment methods, general practitioners in Indonesia have restricted autonomy in prescribing medicines resulting in disparity in the quality of care provided to patients [14]. The equity gap in insurance coverage caused by heterogeneous benefits across different age groups and socioeconomic status magnifies this issue [36].

The three different SHI schemes in Thailand accentuate the issue of inequitable healthcare access as one scheme has expenditure per capita four to five times higher than another scheme [27, 51]. Each scheme offers different healthcare benefits and different levels of access to medication [25]. Inequality between three different schemes impedes progress towards UHC.

#### • Challenges in merging different schemes

Merging different health insurance schemes can be politically challenging as certain groups might lose insurance benefits [35]. In Thailand, for example, civil servants who have the best health insurance benefits resisted a government plan to harmonise the different insurance schemes [51, 52]. Legislative hurdles exist as the three schemes are governed by different legal frameworks. The proposed implementation of an SHI scheme in Malaysia was also met with resistance from civil servants and private sector employees who would have to make mandatory contributions if the proposal came into effect [32].

#### • High administrative cost and loss of negotiating power

Multiple management structures for each insurance scheme incur high administrative costs [25]. More importantly, multiple fragmented health insurance schemes may lead to a loss in negotiating power with health service providers, which is detrimental to cost-containment [35]. The existence of several packages with similar benefits could be streamlined into one and managed by a single authority body. Therefore, duplicating existing benefit packages might also result in redundancy and inefficiency.

#### • Marginalisation of stateless people

As a result of fragmented health insurance schemes as opposed to a unifying and non-discriminatory scheme, vulnerable groups like stateless people who do not possess formal documentation are deprived of their right to access healthcare [30, 31, 40]. Although voluntary health insurance for migrants was introduced in Thailand, it only covered one-third of the 3.4 million migrants [31].

### iii) Incongruity between insurance benefits and people’s need

The factors responsible for the incongruity between insurance benefits and people’s need include the following:

#### • Incomplete insurance coverage

Health insurance in Vietnam excludes medical check-ups and certain technologically advanced treatments [44]. Qualitative evidence indicates the limited use of outpatient services can be due to limited benefit packages [38]. In Thailand, novel and expensive treatment is a barrier to healthcare access due to limited coverage of health insurance schemes [27]. Essential services for non-communicable diseases funded by the Health Equity Fund in Cambodia are not readily available [47]. The inadequate insurance coverage in the Philippines has coerced people experiencing poverty to make OOP if the hospital charges exceed a benefit ceiling [14, 45]. The mismatch in health needs and insurance benefits provided by the SHI hinders progress towards UHC by negatively affecting health-seeking behaviour. High premium rates for maternal health services and dialysis have made these essential treatments inaccessible for people with low incomes in Indonesia [33].

#### • Inequitable distribution of resources

Disparities between different insurance schemes influence healthcare provider payment methods, resulting in limited control over resource allocation by hospitals [27]. As a result, the hospitals’ financial and human resources might be inadequate to meet patients’ demands. The budget for civil servants working at public hospitals in Thailand depends on the hospital size or number of beds [46]. Instead of prioritising hospitals with greater needs, bigger hospitals in more affluent provinces receive more resources. This inequitable distribution of resources is counterproductive towards UHC.

### iv) Political and legislative indifference

The components identified in our study that lead to the political and legislative indifference can be classified as follow:

#### • Conflict of interest between stakeholders

Health financing planning involves various stakeholders with different vested interests. In Malaysia, where the establishment of SHI failed after many years of planning, institutional conflict of interest between the proposed National Health Financing Authority and the Ministry of Health was identified as one of the cardinal factors as there would be a loss of financing power from the latter to the former [32]. There was also resistance from the private sector with concerns about diminishing profits [32].

A similar conflict is also observed in Thailand, with tension between the Ministry of Public Health and the National Health Security Office over allocating public funds [51].

Balancing the interests of the National Health Security Office, civil society and the citizens is also crucial towards UHC achievement [31]. A lack of coherent policies between authorities due to conflicts of interest should be avoided for stability in health financing planning.

#### • Dominant role of politics in policymaking

The political influence over health financing manifests itself in various ways. For example, the yearly enrolment for the Philippines SHI peaked during the election years [32]. The political motivation to improve health financing is blatant, but inconsistencies undermine previous cumulative efforts. The absence of political will to implement SHI in Malaysia is reflected in the inaction despite many years of discussion at the national level [32]. The politicisation of Indonesia’s SHI system impedes progress towards UHC [39], which is exacerbated by the dominant role of politics in policymaking and the lack of medical, economic and financial perspective in the technical operations of SHI [43]. Political instability was also identified as a hindrance towards UHC in Thailand [31].

#### • Lack of legislative framework

In many countries, the absence of legal frameworks to support inclusive coverage for informal sectors contributes to the failure to achieve UHC [37, 54]. In Vietnam, the lack of legal frameworks is indirectly linked to inconsistent policies and results in fluctuating co-payment rates, which particularly impacts the poor [44]. Likewise, poorly regulated private fees due to the laxity of legislation in Malaysia have nocuous effects on the rising OOP trend [32].

### v) Intractable and rapidly rising healthcare cost

From our finding, the following factors explain the intractable nature and rapid growth of healthcare expenditure.

#### • Epidemiological transition and ageing population

The rising cost of healthcare due to the epidemiological transition from communicable diseases to non-communicable diseases (NCD) is observed in several countries [27, 28, 37, 54]. NCDs are generally chronic illnesses that require more healthcare resources. The ageing population is another global trend that can compound healthcare costs in many countries. With the rise in people over 60 years, Thailand is projected to become an ageing society by 2025 [55]. Age-related health problems, like dementia, will translate into increasing healthcare costs while a decline in tax revenue will be observed as the proportion of the working population decreases [48, 55].

#### • Rising demand and rapidly developing health technology

In Indonesia, the population’s growing health awareness and changes in health-seeking behaviour have resulted in rising healthcare demand and associated costs [33]. Medical price inflation is another reason for rising healthcare cost [48]. The thriving pharmaceutical industry has accelerated developments in health technology. Healthcare expenditure has increased in Malaysia due to the procurement of sophisticated medical equipment and novel drugs [28]. Increased expectations for new and costly cancer drugs and increasing demand also contribute to rising healthcare costs [27].

### vi) Morally reprehensible behaviours

Below are the reasons that account for morally reprehensible behaviours in our study:

#### • Insurance-based discrimination

Capitation and diagnosis-related groups are unpopular with the healthcare providers in Thailand due to relatively lower overall payment compared to fee-for-service payment [31]. As a result, there were reports of lower quality of care for patients insured by a certain scheme [27]. Similarly, in Vietnam, insured patients perceived the quality of care received was lower than non-insured members [14, 38]. The capitation system was initially implemented to discourage over-serving, but that has led to discrimination against insured patient [44]. Services to the insured were scaled down to increase the hospital’s revenue. Price discrimination is observed in the Philippines, where users and non-users of SHI are charged different rates by the hospital [45].

#### • Corruptive behaviours

Embezzlement of collected funds for SHI was highlighted as a challenge in Cambodia [50]. In Thailand, attempts of bogus claims reporting comorbidity and complications to increase payment claims for inpatient care were detected [31]. Insurance fraud involving upcoding inpatient conditions for higher reimbursement was also observed in the Philippines [45]. Conversely, actual over-utilisation of healthcare also occurs when unnecessary treatments are provided to increase insurance claims by the health providers [45].

The main themes and synthesized sub-themes are summarised in Fig. 3.

## Discussion

Overall, the studies identified in this systematic review provided invaluable insights into UHC’s health financing challenges. Most of the studies did not set out to directly answer the main objectives of this systematic review. Therefore, it was necessary to apply some inductive reasoning during the data analysis process. Although the study design and methods used in the reviewed studies were not homogenous, this does not undermine their usefulness. The diversity of the studies allowed us to scrutinise the topic from different angles. By distilling the relevant points in these studies, we managed to identify specific challenges to UHC in the ASEAN region, thus addressing the current gap in existing evidence. The following sections describe some potential solutions that can help overcome the identified challenges.

### • Innovative strategies to increase Healthcare Funding

Foreign aid is not a sustainable solution to funding UHC [57]. Expansion of fiscal space with ongoing efforts to optimise the government revenue might be a more pragmatic solution [58]. These have recently been explored in Malaysia, including strategies for increasing government revenue via tobacco and alcohol taxes, and reducing subsidies to foreign users of the public health system [59]. Taxation from lucrative medical tourism could also be implemented to supplement tax revenue [60]. The sustainability of the healthcare finance system can also be approached from the perspective of optimising value generation by the health system via improving service delivery [61]. Regardless, healthcare finance sustainability depends on other essential conditions like long-term economic growth, a stable political environment and strong financial stewardship. There should be a simultaneous effort to improve these complementary aspects.

### • Lessons from other Countries

Fragmented health schemes seem to be a reverberating problem in the ASEAN region. However, countries like the United States also experience many adverse effects from fragmented health schemes and potential benefits could be gained from a unified system [62]. Similar characteristics are observed in Europe, where a single risk pool serves many benefits, like enhancing administrative efficiency and ensuring equitable access to healthcare [61]. Developing countries like Rwanda have also demonstrated difficulty integrating different schemes and proposed that an ideal solution should cover the poorest population and then gradually include other populations [58].

Having one of the oldest SHI systems in the world, Germany offers valuable insights into merging fragmented health schemes. The risk equalisation scheme, which involves redistribution of funds and risks between different insurance schemes [63] could potentially be emulated by Thailand with some modifications. This scheme allows to balance various schemes without threatening the existence of individual schemes and does so without significant tangible effects on the patient’s benefits. The German system has evolved through incremental changes over a long period instead of drastic reform [63]. In countries where compulsory insurance might be politically unfeasible, voluntary schemes may be the next best option [63]. Governments should take a central role in establishing SHI as they are in the best position to create a legal framework.

### • Role of legal Framework

The nascent concept of legal determinants of health has demonstrated the role of law as a catalyst for achieving UHC [64]. Comprehensive national health law is warranted to safeguard the fundamental rights of everyone in the country regardless of income, gender, race and legal residence [65]. Such legal enforcement could potentially curb the marginalisation of stateless people. To support national law reform in various countries, WHO, and United Nations Development Programme and Georgetown University have launched the Legal Solutions for UHC network [65]. Participation in this network could be a first step towards effacing legal indifference in the ASEAN region.

With effective legal enforcement, several other issues could also be addressed. For example, regular auditing with heavy fines to hospitals and healthcare institutions could deter insurance-based discrimination and corruptive behaviour. Such problems are not unique to ASEAN and are also observed in the United States [6, 66]. Furthermore, national legislation can, to some extent, limit the profit margin of newly developed medical technology and regulate the pricing at more affordable levels [65]. However, it is noteworthy that law and regulatory framework serve as quality assurance for care provision and consumer safety in support of UHC within the country itself rather than imposing uniformity in legal framework for health regionally within ASEAN countries.

### • Regional Partnership and collaboration

In light of increasing healthcare costs, stronger emphasis should be placed on health technology assessment (HTA), which focuses on maximising the value of each dollar spent through sound, scientific methods supported by evidence. Collaborative efforts are being made to expand the HTA capacity in the ASEAN region [67] with ongoing an initiative through the ASEAN HTA Harmonization Project. Apart from that, five ASEAN countries (Malaysia, Indonesia, Lao PDR, Vietnam and the Philippines) are part of the Joint Learning Network for Universal Health Coverage (JLN), an innovative, country-driven network of practitioners and policymakers from around the world who collaborate to create global knowledge modules and training to help close the knowledge gap and increase access to healthcare within the population of the participating countries [68]. This systematic review provides information on focus areas to tackle differences and harmonise similarities within and between countries based on the evidence mapping. Drawing from the experience of small island developing states [60], similar solutions might be applicable in different countries with slight adaptations owing to the high degree of similarity in terms of societal setting. ASEAN member states should capitalise on their relative homogeneity in demographics and pool resources to optimise research efficiencies by avoiding duplicating trials and sharing research results. ASEAN countries could benefit from strengthening and strategising the existing partnerships with multilateral international institutions like IMF, World Bank, and OECD.

### • Planning for and mitigating the impact of Ageing Population

Healthcare financing challenges stemming from ageing populations are irrevocable. Recognising the untenable burden on government revenue, Australia innovates private market products specifically for the elderly, like reverse mortgages and life care annuities, to mitigate the economic burden [69]. Actuarial assessment based on estimated probabilities derived from Australian-based individual longitudinal data plays a vital role [69]. ASEAN countries should explore policies on aged care financing integrating retirement income and health financing. Experience from Canada and Japan has proven the usefulness of reforming primary healthcare and curbing the rising healthcare cost associated with ageing populations [70].

### • Capitalising on the benefits of Digitalisation

With the flourishing development of digital technologies, there is vast untapped potential that could provide answers to some of the challenges identified. By maintaining electronic health records, the government-sponsored health insurance program in India has prevented fraud by healthcare providers and patients [71]. The costly administrative fees associated with collecting premium payments in rural areas can also be reduced using digital tools like smartphone applications [71]. Similar solutions would effectively deal with the high prevalence of informal sector workers, whose obscure locations and limited administrative existence can be challenging [71].

Digital technologies also have a role in optimising strategic purchases by data-driven algorithms to perform cost-effectiveness analysis to evaluate the option with the best value [71]. The rapidly growing research in big data and its application in the health sector could potentially reduce healthcare expenditure [72] and healthcare costs [73] in the long run. In the European context, there is increasing application of HTA to navigate decisions relating to reimbursement [61]. Digital reformation should be adopted as a synergistic catalyst towards UHC [74]. The development of a mobile-health wallet application in Kenya known as M-TIBA, which encourages households to set aside money for future unforeseen health expenses, has raised the awareness of preparing for health expenses among the poor [71]. Although it was not designed as insurance, it demonstrated the efficacy of digital technology in initiating a paradigm shift. The benefits of utilising digital technologies for fund collection are (1) the possibility of raising funds outside the formal banking system, and (2) cross-border fund-raising [71]. Nonetheless, concerns like cybersecurity and privacy issues must be addressed.

The complex and diverse nature of health financing challenges requires multi-pronged strategies. The following strategic recommendations should be considered at the national and regional levels.

### Recommendations

*At ASEAN regional level*:

- Strengthening regional collaborative efforts to work towards sustainable UHC-centric policies.
- Developing collective research capacity for cost-effective strategies that focus on ageing populations and transiting demographics.
- Exploring innovative financing schemes, including setting up regional funds which can potentially narrow health and wealth inequality gaps within the region.

*At the national level*:

- Harmonising the national financial strategy towards UHC-oriented goals by strengthening stewardship and engaging all stakeholders.
- Drafting of a national legal framework on healthcare financing which aligns with UHC aims and objectives.
- Investing in digital technologies to reduce healthcare costs and enhance efficiency.

### Limitations

Although the systematic review set out to explore the challenges faced by the ASEAN countries, no articles were found for Brunei, Laos, Singapore or Myanmar specifically. The lack of data among these nations highlights the gap and potential future direction for research. In particular, there is scarcity of data from Singapore and Brunei.

Due to the heterogeneity of articles retrieved, it was not possible to appraise the articles using a single standard checklist. Moreover, quality assessment was omitted for six articles. That would, to some extent, contribute to inconsistency in the quality assessment process.

Finally, the search strategy only included articles published in English, and some challenges published in non-English languages might not be captured in this study.

## Conclusion

This systematic review has identified health financing challenges in the ASEAN region, which stem from a complex mixture of political, economic, moral, legal, epidemiological and bureaucratic factors. These have far-reaching impact on the achievement of UHC, which should be addressed proactively. Addressing these will be highly challenging and requires regional collaborative efforts in nurturing research capacity and exploring innovative financing schemes. National-level initiatives should complement policies that align with UHC objectives, and efforts should be coordinated between the ASEAN nations.

### Electronic supplementary material

Below is the link to the electronic supplementary material.

Additional file 1: Appendix 1. Search strategies used in various databases.

Additional file 2: Appendix 2. Evidence Summary Table.

## Data Availability

The search strategy is attached as a supplementary file. All publications reviewed in this article are listed in the references section. Data sharing is not applicable to this article as no datasets were generated or analysed during the current study.

## Abbreviations

ASEAN: Association of Southeast Asian Nations
CASP: Critical Appraisal Skills Programme
GDP: Gross Domestic Product
HTA: Health Technology Assessment
IMF: International Monetary Fund
JBI: Joanna Briggs Institute
LMIC: Low Middle-income Countries
NCD: Non-communicable Diseases
OECD: Organisation for Economic Co-operation and Development
OOP: Out-of-Pocket Payment
PRISMA: Preferred Reporting Items for Systematic Reviews and Meta-Analyses
SDSN: Sustainable Development Solutions Network
SHI: Social Health Insurance
UHC: Universal Health Coverage
UMIC: Upper Middle-income Countries
WHO: World Health Organisation
