# Supplementary material for: Health financing challenges in Southeast Asian countries for universal health coverage: a systematic review
Source: Arch Public Health. 2023 Aug 17;81:148. doi: 10.1186/s13690-023-01159-3 (PMC10433621; doi:10.1186/s13690-023-01159-3)
Supplement: Supplementary file 1 — Additional file 1: Appendix 1. Search strategies used in various databases. [file 13690_2023_1159_MOESM1_ESM.docx]

**Appendix 1: Search strategies used in various databases**

**Search 1**

Database: CINAHL (Cumulative Index to Nursing & Allied Health)

Platform: EBSCO

Date searched: 22^nd^ June 2022 (Access by University of Glasgow library student account)

| # | Searches | Results |
| --- | --- | --- |
| S1 | TX barriers OR issues OR obstacles OR difficulties OR problems OR challenges OR lessons OR experiences | 1,347,540 |
| S2 | TX health* n3 finance OR health* n3 expenditure OR health* insurance system | 9,500 |
| S3 | TX universal health coverage OR universal health care OR UHC OR universal coverage OR utility OR equity | 85,256 |
| S4 | TX ASEAN OR Southeast asia OR Brunei OR Brunei Darussalam OR Cambodia OR Indonesia OR Lao PDR OR Lao OR Malaysia OR Myanmar OR Singapore Or Thailand OR Vietnam OR Philippines | 97,593 |
| S5 | S1 AND S2 AND S3 AND S4 | 49 |
| S6 | S1 AND S2 AND S3 AND S4  Filter applied “dated from January 2010 onwards”  Limiters  Published Date: 20100101-20221231 | 40 |

**Search 2**

Database: MEDLINE

Platform: OVID

Database coverage: Ovid MEDLINE (R) 1996 to present, In-Process, In-Data-Review and non-indexed citation, Epub ahead of print

Date searched: 22^nd^ June 2022 (Access by University of Glasgow library student account)

| # | Searches | Results |
| --- | --- | --- |
| 1 | (barriers or issues or obstacles or difficulties or problems or challenges or lessons or experiences).mp. | 1760953 |
| 2 | (health* adj3 finance) or (health* adj3 expenditure) or (health* insurance system).mp. | 9013 |
| 3 | (universal health coverage or universal health care or UHC or universal coverage or utility or equity).mp. | 263822 |
| 4 | (ASEAN or Southeast asia or Brunei or Brunei Darussalam or Cambodia or Indonesia or Lao PDR or Lao or Malaysia or Myanmar or Singapore or Thailand or Vietnam or Philippines).mp. | 149485 |
| 5 | 1 and 2 and 3 and 4 | 31 |
| 6 | Applying limit for publication year from 2010 | 27 |

**Search 3**

Database: EMBASE

Platform: OVID

Database coverage: EMBASE 1996 – 2022 week 21, 1946-present

Date searched: 22^nd^ June 2022 (Access by University of Glasgow library student account)

| # | Searches | Results |
| --- | --- | --- |
| 1 | (barriers or issues or obstacles or difficulties or problems or challenges or lessons or experiences).mp. | 2309574 |
| 2 | (health* adj3 finance) or (health* adj3 expenditure) or (health* insurance system).mp | 12816 |
| 3 | (universal health coverage or universal health care or UHC or universal coverage or utility or equity).mp | 377496 |
| 4 | (ASEAN or Southeast asia or Brunei or Brunei Darussalam or Cambodia or Indonesia or Lao PDR or Lao or Malaysia or Myanmar or Singapore or Thailand or Vietnam or Philippines).mp | 198723 |
| 5 | 1 and 2 and 3 and 4 | 37 |
| 6 | 5 + 2010 year limit | 31 |

**Search 4**

Database: PubMed

Date searched: 22^nd^ June 2022

| # | Searches | Results |
| --- | --- | --- |
| 1 | **barriers OR issues OR obstacles OR difficulties OR problems OR challenges OR lessons OR experiences** | 3,920,258 |
| 2 | **health* n3 finance OR health* n3 expenditure OR health* insurance system** | 49,831 |
| 3 | **universal health coverage OR universal health care OR UHC OR universal coverage OR utility OR equity** | 4,138,420 |
| 4 | **ASEAN OR Southeast Asia OR Brunei OR Brunei Darussalam OR Cambodia OR Indonesia OR Lao PDR OR Lao OR Malaysia OR Myanmar OR Singapore Or Thailand OR Vietnam OR Philippines** | 246,857 |
| 5 | 1 and 2 and 3 and 4 | 214 |
| 6 | 5 + filter year 2010 and above | 173 |
| 7 | 6 + article type “review” and “systematic review” only | 23 |

**Search 5**

Database: EconLit

Platform: EBSCO

Date searched: 22^nd^ June 2022 (Access by University of Glasgow library student account)

| # | Searches | Results |
| --- | --- | --- |
| S1 | TX barriers OR issues OR obstacles OR difficulties OR problems OR challenges OR lessons OR experiences | 413,670 |
| S2 | TX health* n3 finance OR health* n3 expenditure OR health* insurance system | 10,366 |
| S3 | TX universal health coverage OR universal health care OR UHC OR universal coverage OR utility OR equity | 130,297 |
| S4 | TX ASEAN OR Southeast asia OR Brunei OR Brunei Darussalam OR Cambodia OR Indonesia OR Lao PDR OR Lao OR Malaysia OR Myanmar OR Singapore Or Thailand OR Vietnam OR Philippines | 52,476 |
| S5 | S1 AND S2 AND S3 AND S4 | 46 |
| S6 | S5 and **Limiters** - Published Date: 20100101-20221231 | 39 |
