# Supplementary material for: Health financing challenges in Southeast Asian countries for universal health coverage: a systematic review
Source: Arch Public Health. 2023 Aug 17;81:148. doi: 10.1186/s13690-023-01159-3 (PMC10433621; doi:10.1186/s13690-023-01159-3)
Supplement: Supplementary file 2 — Additional file 2: Appendix 2. Evidence Summary Table. [file 13690_2023_1159_MOESM2_ESM.docx]

| **No** | **Journal**  **Year** | **First Author** | **Country discussed / scope covered** | **Health-financing Challenges related to UHC** |
| --- | --- | --- | --- | --- |
| 1 | PLoS One  2019 | Chaw-Yin Myint | ASEAN in general | - Fragmented health insurance scheme - Low level of government spending - Low government revenue - Rising healthcare cost and usage threatening health system financial stability |
| 2 | Health Policy and Planning  2012 | Bart Jacobs | ASEAN in general | - Inability to generate cash for unforeseen healthcare needs - Supply side determined prices leading to inability to afford healthcare |
| 3 | BMC Public Health  2020 | Wenhui Mao | Vietnam | - Problem with defining and reaching out to poor population - Unable to design effective cross subsidization mechanism - Difficult to ensure sustainability of health insurance schemes - Rising cost of health expenditure due to epidemiological transition |
| 4 | Review of Development Economic  2019 | Midori Matsushima | Vietnam | - Corruptive behaviour hindering the implementation health financing process - Health insurance scheme not serving its purpose - Incomplete insurance coverage, no medical check-up meaning higher risk of advance disease during the diagnosis stage - Inconsistent health financing policy with fluctuating co-payment rates - Insufficient payment of staff in public practice |
| 5 | Journal of Public Health  2019 | Nel Jason L. Haw | Philippines | - Moral hazard issues relating to health financing - Rising health care cost as a health financing challenge - Failure of health insurance scheme to achieve its goal of financial risk protection |
| 6 | Health Policy and Planning  2020 | Fatim Lakha | Thailand | - High cost of certain novel and expensive treatment as a barrier to healthcare access due to limitations of health insurance scheme - Imbalance in equitable access due to different entitlements for the health insurance schemes - Lack of political will to create a regulatory environment that contributes to long term success in UHC health financing in terms of potential benefits gained by research infrastructure investment and capacity building - Question of sustainability of tax financed policy - Resistance from health service providers for cost containment by curtailing fee-for-service payment - Rising cost of healthcare due to epidemiological transition - Rising healthcare cost as a result to increased expectations for new and expensive intervention |
| 7 | International Journal for Equity in Health  2012 | Xiaoyun Liu | Vietnam | - Development of common social health insurance model can be time consuming leading in delayed attainment in UHC - Incomprehensive coverage of health insurance coverage due to limited benefit package and discrimination against health insurance members - Long waiting period for receiving health insurance cards because of administrative inefficiency - Perception of disparity between healthcare quality received between members and non-membersSubstandard care provided to insured member |
| 8 | Human Resources for Health  2015 | Wilailuk Ruangratanatrai | Thailand | - Declining government funding - Government policy that is in disharmony with necessary changes in UHC reform health policies - Health financing for human resources becoming less pro-poor. inequity in health financing - Inequity in healthcare distribution in human resource financing (as part of health financing) - Lack of political will to create changes in favour for UHC goals - Rising cost of healthcare causing a burden on health financing system |
| 9 | BMC Public Health  2011 | Hong Teck Chua | Malaysia | - Expanding private healthcare posing risk to health equity and universal coverage - Health financing challenged by the lack of allocative, technical and distributional efficiency - Health financing policies did not address the national health needs, especially the poor and preventive and promotive. - Lack of focus on cost-effectiveness - Lack of government and political support for health financing to achieve these UHC goals - Lack of government will to accelerate development of prepayment financing mechanism in the form of a social health insurance - Problem of sustainability of health financing plagued by epidemiological transition --> increasing NCD and associated spending - Rising health care cost due to high end medical equipment and new drugs - System disparity between public and private healthcare might threaten the three dimension of UHC as it starts to expand its coverage |
| 10 | Applied Health Economics and Health Policy  2015 | Kannika Damrongplasit | Thailand | - Chronic underfunding leading to compromise of quality of care - Discrepancies in amount of healthcare funding requested versus amount of funding actually received - Sustainability of health financing system is questionable with rising healthcare cost coupled with slowdown in overall economic growth. |
| 11 | Global Health Action  2017 | Celia McMichael | ASEAN in general,  Thailand | - General: Migrants are not included in health insurance schemes, migrant health programmes are underfunded. - Thailand: Migrant workers’ pre-employment insurance only allows them to seek healthcare from certain hospital |
| 12 | Health Policy and Planning  2021 | Theepakorn Jithitikulchai | Cambodia | - Incomprehensive coverage of the services by the HEF fund resulting in suboptimal utilisation - Ineffectiveness of Health Equity Funding to achieve its UHC related goals of increasing public health service utilisation |
| 13 | The Lancet  2018 | Viroj Tangcharoensathien | Thailand | - Conflict of interest exist between different stakeholders which might jeopardise the overall achievement towards UHC - Delay in achievement of UHC because health finance contributory scheme requires administrative capacity due to large size of informal sector in developing countries. - Difficulty in deciding method to finance uninsured population, between voluntary contribution and general taxation. - Ensuring sustainable health financing policies under the backdrop of epidemiological transition with rising healthcare cost - Failing to address the funding issue for non-Thai citizens - Falsely reporting to increase payment for inpatient care resulting in unnecessary additional expenditure - Political instability as a hurdle to UHC financing model - Termination of supply side financing leading to conflict between stakeholders with conflicting interest |
| 14 | The Lancet  2011 | Viroj Tangcharoensathien | ASEAN in general, Cambodia, Laos, Malaysia,  Philippines, Thailand, Vietnam | - General:   - Difficulty in identifying the poor in many countries posing as a challenge to health-financing scheme candidate screening   - High percentage of uninsured population leading to risk of financial impoverishment for many   - Insufficient government funding allocation for health   - Lack of suitably developed insurance scheme to cover the informal sector, ie non-poor and not-so-poor   - Low total health expenditure per capita, lower than necessary required for meeting the MDG - Cambodia:   - Co-payment acting as a barrier to healthcare access for the poor   - Sustainability of the health financing system - Laos: Insufficient government funding - Cambodia and Laos: Funding via external donors impacting the long-term sustainability of health financing system - Malaysia:   - Difficulty in premium collection from informal sector   - High level of OOP from private healthcare   - Lack of social health insurance   - Poorly regulated private fees   - Potential occurrence of institutional conflict of interest with establishment of social health insurance scheme   - Rising health care cost due to epidemiological transition   - The lack of political will as a hurdle to implementation of national social health insurance - Philippines and Indonesia: Difficult in decentralised system to mobilise political will to improve financial commitment to the poor and vulnerable. - Philippines:   - Adverse selection within the scheme as most members enrolled are chronically-ill and has high rate of us   - Difficulty in collection of premium for social insurance in the form of expensive administrative cost and mobility of members   - Hard to reach informal sector   - Inadequate coverage of social health insurance schemes leading to inadequate financial protection   - Inconsistency of effort in promoting UHC related schemes, peaking during election years, politically motivated rather than needs based   - Insufficient coverage for poor due to lack of local govt financial commitments - Thailand:   - Financial feasibility   - Political hindrance   - Technical problem with premium collection in informal sector - Vietnam:   - Challenging to collect premium in rural areas, high administrative cost   - Challenging to cover the informal sector   - Ineffective health financing policy that failed to have impact on poorest population |
| 15 | International Journal for Equity in Health  2020 | Haruyo Nakamura | Cambodia | - Embezzlement problem during premium collection - High administrative cost associated with premium collection - Unwillingness to contribute to social health insurance due to low level of awareness |
| 16 | Health Policy and Planning  2017 | Elizabeth Pisani | Indonesia | - Political influence on the development on UHC health financing methods |
| 17 | The Journal of Health Care Organisation, Provision and Financing  2020 | Manushi Sharma | Indonesia | - High premium insurance for certain services creating a barrier for access for certain treatment like cancer treatment and dialysis - Insufficient spending per capita on health - Unregulated health care demand overwhelming the budget available resulting in large financial deficit |
| 18 | International Journal of Social Economics  2017 | Ravikan Nonkhuntod | Thailand | - Differences in payment system resulting in discriminatory treatment leading to less than ideal utilisation of health care - Fragmented schemes causing redundancy and increased administrative cost amounting to inefficiency - Question of long term sustainability and quality of healthcare if funding is solely reliant on government funding |
| 19 | Global Social Policy  2020 | Somsak Chunharas | Thailand | - Conflict of interest due to establishment of new authority that undermine the autonomy in fund allocation - Attempt to harmonise fragmented insurance schemes raising resistance from civil servants who had the best funded public scheme - Question of long term sustainability with tax-financed budget |
| 20 | Social Science & Medicine  2011 | Cheng Li | Thailand | - Presence of large informal sector makes it difficult for premium collection |
| 21 | Bulletin of World Health Organisation  2019 | Viroj Tangcharoensathien | Thailand | - Budget decision did not lie on ministry of health but finance ministry - Inadequate budget allocation for healthcare - Presence of adverse selection for voluntary health insurance scheme because more sick people joined than healthy people - Presence of informal sector is a major barrier in achieving UHC - Resistance from different strata of the society regarding financial reforms as certain groups might be disadvantaged |
| 22 | The Economics of Peace and Security Journal  2020 | Chantal Herberholz | Thailand | - Stateless people not covered by health insurance thus undermining UHC achievement |
| 23 | Asia-Pacific Development Journal  2011 | Giang Thanh Long | Vietnam | - Challenging to change people's mindset towards health insurance, - Presence of large informal sector and non-working family - Health insurance not fulfilling its function, health insurance holder from vulnerable groups has low accessibility - Discriminatory attitude towards those who hold insurance cards - Low income, poor quality of public service, poor marketing, poor flexibility in payment causing low uptake of voluntary insurance scheme - Presence of regional inequalities, both horizontal and vertical, in state's allocation funding for health insurance. |
| 24 | Social Science & Medicine  2015 | Minchung Hsu | ASEAN in general,  Thailand | - General: Rapidly rising medical price inflation - Thailand:   - Long term sustainability of the financing system   - Presence of large informal sector and rapid population aging   - Rapid aging population increases health care cost and informal sector reduces ability to raise tax revenue |
| 25 | International Journal for Equity in Health  2021 | Mohammad Bazyar | ASEAN in general, Indonesia, Thailand | - General: Fragmentation of health insurance reduces potential degree of redistribution, high administrative costs, duplication of benefits, loss of negotiation power with health services providers - Indonesia:   - Employers worrying that premiums paid would be used to subsidise services for poor and unwaged   - Fragmented health financing system, decentralisation, demographic transition, high out-of-pocket spending, low level of spending on health   - Low enrolment of national health insurance scheme because of informal sector workers   - Low enrolment rate jeopardising the long term financial stability of national health insurance   - Resistance from private employers to join national health insurance worrying that contribution rates might increase - Thailand: Inequality between the three different health insurance schemes |
| 26 | National Public Health Journal  2021 | Atikah Adyas | ASEAN in general, Indonesia, Myanmar | - General: Lack of legal framework to cover the informal sector and lack of political will - Indonesia:   - Gap exist between enrolment and actual utilisation of health facilities owing to lack of knowledge   - Epidemiological transition in the form of rising NCDs putting pressure on health budget and NHI - Myanmar and Indonesia: Unequal presence of health service providers as a challenge to providing insurance |
| 27 | Global Health Action  2018 | Konrad Obermann | Philippines | - Lack of long term sustainability due to source of budget from a non-governmental third party source |
| 28 | Global Public Health  2022 | Hien Thi Ho | Vietnam | - Cumbersome reimbursement process deterring members to join FHI - FHI not affordable - Informal sector workers feel that insurance is more relevant for older people as they are more at risk, while young people report other financial priorities - Perception of low quality of healthcare service provided by FHI - Presence of adverse selection, only purchasing when a family member or themselves are ill - Presence of large proportion of informal sector workers falling through gaps of UHC financing due to lack of formal salary or taxation |
| 29 | Health Policy and Planning  2020 | Erniaty Erniaty | Indonesia | - Political forces fail to consider the technical operations from financial perspective |
| 30 | Bulletin of World Health Organisation  2019 | Kanitsorn Sumiriddetchkajorn | Thailand | - Ageing population contributing to rising health care cost - Fragmented insurance schemes resulting in disparity in healthcare access - NCD contributing to rising healthcare cost - No unifying mechanism to control expenditure under three fragmented schemes - Question of long term sustainability with tax financed policy - Rising healthcare cost with expectation of population and increasing cost of technology |
| 31 | The Lancet  2019 | Rina Agustina | Indonesia | - Equity gap in insurance coverage - Inadequate government spending on health expenditure - Members not paying their contribution routinely - Presence of large informal sector worker makes it challenging to implement health care insurance system |
| 32 | Applied Health Economics and Health Policy  2020 | Teguh Dartanto | ASEAN in general, Indonesia | - General:   - Health utilisation decreasing affecting premium payment   - Lack of health literacy leading to increased premium payment dropout rates   - Perception of poor quality of healthcare resulting in dropout from health insurance - Indonesia:   - Administrative difficulty in collecting contribution in cost effective manner among informal workers   - Failure of NHIS from raising revenue resulting in question in long term sustainability   - Other financial hardship making household to prioritise other essential expenditures over paying for premium insurance   - Premium cost for insurance too expensive |
